# Prevalence of adequate postnatal care and associated factors in Rwanda: evidence from the Rwanda demographic health survey 2020

**DOI:** 10.1186/s13690-022-00964-6

**Published:** 2022-09-16

**Authors:** Joseph Kawuki, Ghislaine Gatasi, Quraish Sserwanja

**Affiliations:** 1 Centre for Health Behaviours Research, Jockey Club School of Public Health and Primary Care, The Chinese University of Hong Kong, Hong Kong, SAR China; 2 Key Laboratory of Environmental Medicine Engineering, School of Public Health, Southeast University, Nanjing, 210009 Jiangsu Province China; 3 Programmes Department, GOAL, Arkaweet Block 65 House No. 227, Khartoum, Sudan

**Keywords:** Postnatal care, Women, Rwanda, DHS, Adequate, Quality

## Abstract

**Background:**

Although quality postnatal care (PNC) is a known significant intervention for curbing maternal and newborn morbidity and mortality, it is underutilized in most developing countries including Rwanda. Thus, it is crucial to identify factors that facilitate or occlude receipt of adequate PNC. This study aimed at assessing the prevalence of adequate PNC content and the associated factors in Rwanda.

**Methods:**

We used weighted data from the Rwanda Demographic and Health Survey (RDHS) of 2020, comprising of 4456 women aged 15–49 years, who were selected using multistage sampling. Adequate PNC was considered if a woman had received all of the five components; having the cord examined, temperature of the baby measured, counselling on newborn danger signs, counselling on breastfeeding and having an observed breastfeeding session. We, then, conducted multivariable logistic regression to explore the associated factors, using SPSS version 25.

**Results:**

Out of the 4456 women, 1974 (44.3, 95% confidence interval (CI): 43.0–45.9) had received all the PNC components. Having no radio exposure (adjusted odds ratio (AOR) =1.41, 95% CI: 1.18–1.68), visited by a fieldworker (AOR = 1.35, 95% CI: 1.16–1.57), no big problem with distance to a health facility (AOR = 1.50, 95% CI:1.24–1.81), and residing in the Southern region (AOR = 1.75, 95% CI: 1.42–2.15) were associated with higher odds of adequate PNC compared to their respective counterparts. However, having no exposure to newspapers/magazines (AOR = 0.74, 95% CI: 0.61–0.89), parity of less than 2 (AOR = 0.67, 95% CI: 0.51–0.86), being a working mother (AOR = 0.73, 95% CI: 0.62–0.85), no big problem with permission to seek healthcare (AOR = 0.54, 95% CI: 0.36–0.82), antenatal care (ANC) frequency of less than 4 times (AOR = 0.79, 95% CI: 0.62–0.85), inadequate ANC quality (AOR = 0.56, 95% CI: 0.46–0.68), and getting ANC in a public facility (AOR = 0.57, 95% CI: 0.38–0.85) were associated with lower odds of adequate PNC.

**Conclusions:**

Less than half of the mothers in Rwanda had received adequate PNC, and this was associated with various factors. The results, thus, suggested context-specific evidence for consideration when rethinking policies to improve adequate PNC, including a need for intensified PNC education and counselling during ANC visits, continued medical education and training of PNC providers, and strengthening of maternal leave policies for working mothers.

**Supplementary Information:**

The online version contains supplementary material available at 10.1186/s13690-022-00964-6.

## Background

The levels of maternal and child mortality are often used to evaluate a country’s health system’s performance, and the high rates of which have consistently been one of the most serious public health challenges and still exist in some areas to date [1, 2]. Despite the rise of maternal and child programs and global maternal health communities in the most affected countries, the risk of both the mother and the newborn dying after childbirth remains high [1, 2]. As a result, there is a growing body of evidence and recommendations emphasizing the importance of delivering high-quality, and cost-effective care throughout this period [3, 4].

The postpartum period is essential, with implications for both the mother and newborn child’s health and survival [5]. Increased morbidity and death are typically the results of a lack of appropriate, suitable, or timely care during that period [5]. Indeed, more than half of neonatal deaths occur within the first 2 days after birth, and three quarters occur within the first week [6, 7]. Similarly, 45% of postpartum maternal deaths occur within 24 hours of giving birth, and the risk persists into the second week [8].

Preterm delivery problems, birth asphyxia, and sepsis account for more than 75% of all neonatal deaths, while postpartum haemorrhage, hypertensive disorders, and infections account for the majority of maternal deaths [6]. These are all avoidable and manageable causes which suggest that high-quality care during labour and delivery, as well as skilled care and therapy in the early postpartum period, might help to reduce this fatality [9]. Therefore, global efforts have been directed in that area. Postnatal care (PNC), which consists of a set of services offered to the mother and newborn beginning immediately after the placenta is delivered and continuing throughout the first 42 days of life [10, 11], is one of the most significant programs for improving mother and child health. Postnatal visits are indeed an opportunity for healthcare providers to promote healthy behaviours such as breastfeeding, adequate cord cleaning and handwashing, assess for danger signs and monitor the mother’s and newborn’s recovery, growth, and overall health [5]. It also enables early detection and treatment of childbirth-related problems, as well as counselling and referral to advanced care whenever needed [5].

Geographic disparities in child and mother survival are significant; while global mortality rates have been declining, low and middle-income countries, especially those in sub-Saharan Africa, continue to account for the majority of maternal deaths as well as the highest neonatal death rates [12]. Rwanda is a country in Eastern Africa’s Great Lakes Region that is subdivided administratively into Kigali City and four provinces (Northern, Southern, Eastern and Western) [13]. It is one of the priority countries that has successfully reduced maternal and neonatal mortality based on the previous Millennium Development Goals (MDGs) [14], and this followed the improvement and recovery of the political and economic situation in the aftermath of the civil unrest that marked the early 1990s [15–17]. These achievements were a result of strong government support for key maternal and child health interventions aimed at increasing PNC uptake, such as increasing healthcare providers’ capacity in emergency obstetric and new-born care, connecting women and neonates in need of care to community health workers (CHWs), using community performance-based financing to motivate CHWs to support mothers and babies, and providing free maternal services as well as incentives [13, 18]. However, like most countries globally, Rwanda lagged in ensuring reduction of neonatal mortality with a neonatal mortality rate (NMR) currently at 19 deaths per 1000 live births and contributing over 42% of under 5 mortality [19, 20]. Nonetheless, the country registered great improvement in the maternal mortality ratio (MMR) between 2005, 2010 and 2015 from 750 deaths per 100,000 live births to 210 deaths per 100,000 live births but a slow pattern was registered between 2015 and 2020 from 210 deaths per 100,000 live births to just 203 deaths per 100,000 live births [20].

Despite the considerable outcomes and the multiple benefits of PNC in lowering maternal, neonatal and child mortality, postnatal care service usage in Rwanda remains below the global targets of currently 70% [20]. Regardless of where childbirth takes place, the World Health Organization normally recommends that all mothers and newborns receive their first postnatal assessment within the first 24 hours, followed by their second and third checkups between days 3–4, and between days 7–14, respectively; finally, at around 6 weeks postpartum, a fourth visit completes the recommended four-visits schedule [21]. Besides the number of visits, providing adequate PNC content is absolutely essential in minimizing avoidable deaths in the postpartum period. Existing research in Rwanda has largely focused on the factors that influence PNC visits, such as knowledge, access, and other barriers [13, 22, 23], with little emphasis on the PNC content provided. Based on data from the 2020 Rwanda Demographic and Health Survey (RDHS), this study aimed at assessing the quality of PNC content and the associated socio-demographics in Rwanda. Nevertheless, we hypothesized that several socio-demographic factors determine the utilisation of adequate PNC. Having a better understanding of such factors that influence adherence to or failure to utilize the recommended PNC contents is vital in informing and guiding policy on how to overcome these specific barriers.

## Methods

### Study sampling and participants

The 2019–20 Rwanda Demographic Survey (RDHS) was used for this analysis and employed a two-stage sample design with the first stage involving sample points (clusters) selection consisting of enumeration areas (EAs) [20]. The second stage involved systematic sampling of households in all the selected EAs leading to a total of 13,005 households [20]. The data used in this analysis were from the household and the woman’s questionnaires.

Data collection started in November 2019 and ended in July 2020 taking longer than expected due to the COVID-19 pandemic restrictions [20]. Women aged 15–49 years who were either permanent residents of the selected households or visitors who stayed in the household the night before the survey were eligible to be interviewed. Out of the total 13,005 households that were selected for the survey, 12,951 were occupied and 12,949 were successfully interviewed leading to a 99.9% response rate [20]. This study included women who had given birth within 5 years preceding the survey and had at least one postnatal check whether before discharge from health facility after birth or after home delivery/discharge from the health facility. Among the interviewed households, 14,675 women aged 15–49 were eligible to be interviewed and 14,634 women were successfully interviewed leading to a 99.7% response rate [20]. Out of the 14,634 successfully interviewed women, a weighted sample of 6302 women had given birth within the last 5 years preceding the survey and 4456 had had at least one postnatal check.

### Variables

#### Dependent variables

The outcome variable was the content of postnatal care (PNC). Based on the WHO recommendations [24] and availability of data in the 2020 RDHS dataset, adequate content of PNC was considered when a woman was able to have received all the five PNC components that included: having the cord examined, temperature of the baby measured, counselling on newborn danger signs, counselling on breastfeeding and having had an observed breastfeeding session [25]. This information was self-reported by the women.

#### Independent variables

Andersen’s behavioral model of health service use was adapted considering data availability and evidence from available literature [13, 20, 25, 26] to examine the factors associated with utilization of adequate PNC, as shown in Table 1. As per Andersen’s behavioral model, utilization of healthcare is a function of three major elements: predisposing factors, enabling factors and healthcare needs [29]. The predisposing factors in the model were: age, level of education, region of residence, place of residence, religion, marital status, household size, and parity. Wealth index, working status, having health insurance, exposure to mass media, being visited by a field health worker, seeking permission and distance to the nearest health facility as an indicator of access were considered as enabling factors, while place of childbirth and ANC, ANC frequency and quality were included in the model as a proxy for the need factor [28], as illustrated in Supplementary file 1.

**Table 1:** Categorization of independent variables as obtained from the 2020 Rwanda Demographic Health Survey dataset

Variable	Categorization	Remarks/ description
Exposure to newspapers or magazines	No and Yes	Yes, included those exposed to less than once and at least once a week and No included all those that reported no exposure to newspapers/magazines.
Exposure to radio	No and Yes	Yes, included those exposed to less than once and at least once a week and No included all those that reported no exposure to radio.
Exposure to television (TV)	No and Yes	Yes, included those exposed to less than once and at least once a week and No included all those that reported no exposure to TV.
Access to internet	Yes and no	This was self-reported with yes for those who reported using internet while no for those who were not using internet
Age	15 to 24 years, 25 to 34 years and 35 to 49 years	This was a continuous variable that was later categorized.
Residence	Urban and Rural	
Region	North, East, South, West and Kigali	
Household size	Less than 6 and above 6	Based on the dataset average of 5.2
Parity	1, 2–4 and 5 and above	This was a continuous variable that was later categorized.
level of education	No education, primary, secondary, and tertiary	
Working status	Yes and no	
Wealth index	Richest, richer, middle, poorer, and poorest quintiles	Wealth index is a measure of relative household economic status and was calculated by DHS from information on household asset ownership using Principal Component Analysis [20], which was further categorized into poorest, poorer, middle, richer and richest quintiles.
Having health insurance	Yes and no	
Having been visited by a field health worker within the last 12 months	Yes and no	
Problems seeking permission to go to a health facility	No big problems and big problems	RDHS had three original self-reported categories (no problem, no big problem and big problem) however, after data collection, no woman reported no problem
Problems with distance to the nearest health facility	No big problems and big problems	RDHS had three original self-reported categories (no problem, no big problem and big problem) however, after data collection, no woman reported no problem.
Marital status	Married and not married	Married included both formal and informal unions and not-married included all women that were not in formal or informal unions.
Place of ANC	Private and public health facilities	Private facilities included polyclinics, clinics, and dispensaries while public included referral and district hospitals, health centers, posts, and outreaches. These were combined due to the limited numbers in each sub-category.
ANC quality	Adequate and inadequate	Adequate care was defined as having received all the available six components of ANC that included; having blood pressure measurement, urine, blood samples being taken, being given iron tablets/syrups, intestinal parasite drugs and having had at least two tetanus injections while inadequate was defined as having less than 6 components [27].
ANC frequency	4 and above contacts and less than 4 contacts	4 and above included all women who had utilized at least 4 ANC contacts and less 4 included those who has less than 4 ANC contacts.
Place of childbirth	Home and health facility	Home birth included all women who had their childbirth at home/outside health facility while health facility birth included all births that occurred in a health facility [28].

### Statistical analysis

In order to account for the unequal probability sampling in different strata [30] and to ensure representativeness of the study results [31], DHS sample weights were applied. We used SPSS (version 25.0) statistical software complex samples package incorporating the following variables in the analysis plan to account for the multistage sample design inherent in the DHS dataset: individual sample weight, sample strata for sampling errors/design, and cluster number [32–34]. Initially, we did descriptive statistics for both dependent and independent variables. Frequencies and proportions/percentages for categorical dependent and independent variables have been presented. Afterwards, bivariable logistic regression was done to assess the association of each independent variable with adequate content of postnatal care and crude odds ratio (COR), 95% confidence interval (CI) and *p*-values are presented. Independent variables found significant at bivariable level with *p*-values less than 0.25 were added in the multivariable logistic regression model. Hosmer and Lemeshow test was finally done to test the goodness of the multivariable regression model. Adjusted odds ratios (AOR), 95% Confidence Intervals (CI) and *p*-values were calculated at significance level of 0.05 [35]. All variables in the model were assessed for multi-collinearity, which was considered present if the variables had a variance inflation factor (VIF) greater than 2.5 [36].

## Results

**Fig. 1:**
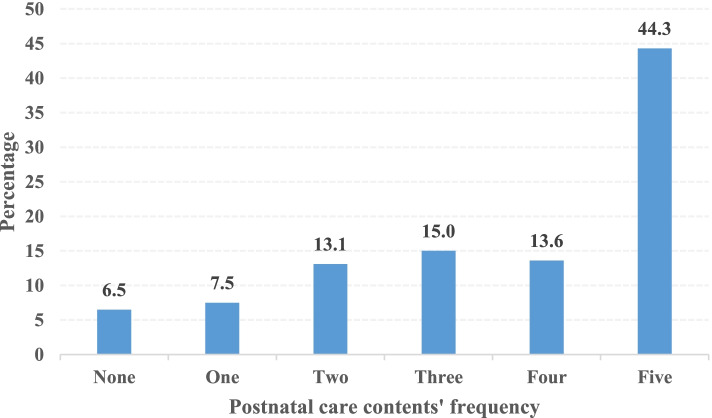
Percentage distribution of postnatal care content utilization frequency as per 2020 Rwanda Demographic Health Survey

A total of 4456 women were included in the analysis (Table 2). The majority of women were married (80.9%), had primary education (64.3%), had no internet access (88.0%), had health insurance (83.2%), were working (75.6%), resided in rural areas (81.7%), had 4 and above ANC contacts (50.8%) and had received inadequate ANC content (84.7%). Regarding PNC content, out of the 4456 women, 1974 (44.3, 95% CI: 43.0–45.9) had received all the postnatal care components, of which the five components scored as follows; had the cord examined (73%), temperature of the baby measured (59.8%), counselling on newborn danger signs (55.4%), counselled on breastfeeding (79%) and had breastfeeding session observed (87.3%), as shown in Table 3. The percentage distribution of postnatal care content utilization frequency is shown in Fig. 1.

**Table 2:** Sociodemographic characteristics of women who received at least one postnatal check in the 2020 Rwanda Demographic Health Survey

Characteristics	***N*** = 4456 (%)
**Age**	
35 to 49	1514 (34.0)
25 to 34	2163 (48.5)
15 to 24	779 (17.5)
**Education Level**	
No Education	432 (9.7)
Primary Education	2864 (64.3)
Secondary Education	940 (21.1)
Tertiary	220 (4.9)
**Exposure to newspapers/magazines**	
No	3474 (78.0)
Yes	982 (22.0)
**Exposure to radio**	
No	903 (20.3)
Yes	3553 (79.7)
**Exposure to TV**	
No	2517 (56.6)
Yes	1939 (43.4)
**Internet access**	
No	3920 (88.0)
Yes	536 (12.0)
**Parity**	
1	1168 (26.2)
2–4	2551 (57.3)
5 and above	737 (16.5)
**Marital**	
Not married	852 (19.1)
Married	3604 (80.9)
**Has health insurance**	
No	747 (16.8)
Yes	3709 (83.2)
**Visited by a fieldworker**	
No	2740 (61.5)
Yes	1716 (38.5)
**Working status**	
Not working	1086 (24.4)
Working	3370 (75.6)
**Permission to access healthcare**	
Big problem	140 (3.2)
Not big problem	4316 (96.8)
**Distance to a health facility**	
Big problem	971 (21.8)
Not big problem	3485 (78.2)
**Residence**	
Rural	3639 (81.7)
Urban	817 (18.3)
**Region**	
North	680 (15.3)
East	1338 (30.0)
West	825 (18.5)
South	970 (21.8)
Kigali	643 (14.4)
**Wealth Index**	
Poorest	943 (21.2)
Poorer	827 (18.6)
Middle	871 (19.5)
Richer	929 (20.8)
Richest	887 (19.9)
**ANC quality**	
Adequate	683 (15.3)
Inadequate	3773 (84.7)
**ANC frequency**	
4 and above	2265 (50.8)
Less than 4	2191 (49.2)
**Place of childbirth**	
Home	6 (0.1)
Health facility	4450 (99.9)
**ANC facility**	
Private	172 (3.9)
Public	4284 (96.1)

*ANC* Antenatal care

**Table 3:** Postnatal care content utilization as per the 2020 Rwanda Demographic Health Survey

Postnatal care service	Frequency, ***N*** = 4456 (%)
Cord examined	3257 (73.1)
Temperature measured	2664 (59.8)
Counselled on newborn dangers	2467 (55.4)
Counselled on breastfeeding	3522 (79.0)
Breastfeeding session observed	3891 (87.3)

### Factors associated with adequate PNC content

Factors found significantly associated with adequate PNC on bivariable logistic regression are detailed in Table 4. After executing multivariable logistic regression controlling for all included variables, the factors that remained significant include; exposure to newspapers/magazines, exposure to radio, parity, working status, visited by a fieldworker, permission to seek health care, distance to a health facility, region, ANC frequency, ANC quality, and ANC facility.

**Table 4:** Factors associated with adequate content of postnatal care in Rwanda as per the 2020 Rwanda Demographic Health Survey

Characteristics	Crude odds ratio (COR) COR (95% CI)	***P***-value	Adjusted odds ratio (AOR) AOR (95% CI)
**Age**			
35 to 49	1		1
25 to 34	0.93 (0.81–1.08)	0.347	1.00 (0.84–1.19)
15 to 24	**0.81 (0.67–0.98)**	**0.034**	0.97 (0.75–1.25)
**Exposure to newspapers/magazines**			
Yes	1		1
No	**0.70 (0.60–0.82)**	**< 0.001**	**0.74 (0.61–0.89)**
**Exposure to radio**			
Yes	1		1
No	**1.20 (1.02–1.40)**	**0.029**	**1.41 (1.18–1.68)**
**Exposure to TV**			
Yes	1		1
No	**0.84 (0.73–0.96) (0.98–1.35)**	**0.013**	0.86 (0.73–1.02)
**ANC quality**			
Adequate	**1**		**1**
Inadequate	**0.55 (0.46–0.66)**	**< 0.001**	**0.56 (0.46–0.68)**
**Parity**			
5 and above	**1**		**1**
2–4	0.90 (0.75–1.07)	0.217	0.83 (0.67–1.02)
Less than 2	0.80 (0.65–0.99)	**0.036**	**0.67 (0.51–0.86)**
**Working**			
No	**1**		**1**
Yes	**0.73 (0.62–0.85)**	**< 0.001**	**0.73 (0.62–0.85)**
**Visited by a fieldworker**			
No	**1**		**1**
Yes	**1.50 (1.30–1.74)**	**< 0.001**	**1.35 (1.16–1.57)**
**Permission to access healthcare**			
Big problem	**1**		**1**
Not big problem	**0.61 (0.41–0.93)**	**0.020**	**0.54 (0.36–0.82)**
**Distance to a health facility**			
Big problem	**1**		**1**
Not big problem	**1.47 (1.23–1.77)**	**< 0.001**	**1.50 (1.24–1.81)**
**Region**			
North	1		1
East	1.13 (0.91–1.39)	0.269	1.17 (0.94–1.45)
West	**1.35 (1.09–1.67)**	**0.006**	1.24 (0.99–1.54)
South	**1.89 (1.55–2.30)**	**< 0.001**	**1.75 (1.42–2.15)**
Kigali	1.07 (0.82–1.40)	0.626	0.88 (0.66–1.18)
**ANC frequency**			
4 and above	1		1
Less than 4	**0.74 (0.64–0.84)**	**< 0.001**	**0.79 (0.69–0.91)**
**ANC facility**			
Private	1		1
Public	**0.60 (0.41–0.88)**	**0.008**	**0.57 (0.38–0.85)**

Bold: significant at < 0.05, *RDHS* Rwanda Demographic Health Survey

The attainment of adequate PNC is smaller in women not exposed to newspapers/magazines compared to their exposed counterparts (AOR= 0.74, 95%CI: 0.61–0.89), while women with no radio exposure had 1.41 (95%CI: 1.18–1.68) higher odds of attaining adequate PNC compared to those exposed to radio. Women with parity of less than 2 had smaller odds of attaining adequate PNC compared to those with parity of 5 and above (AOR 0.67, 95%CI: 0.51–0.86)).

Working women had smaller odds (AOR= 0.73, 95%CI: 0.62–0.85) of having adequate PNC compared to non-working counterparts, unlike those visited by a fieldworker, who had (AOR 1.35, 95%CI: 1.16–1.57) higher odds compared to those not visited by a fieldworker. Moreover, compared to their respective counterparts, women with no big problem with permission to seek healthcare had smaller odds of attaining adequate PNC (AOR = 0.54, 95%CI: 0.36–0.82), unlike those having no big problem with distance to a health facility who had (AOR= 1.50, 95%CI: 1.24–1.81) higher odds of attaining adequate PNC. Compared with women in the North, those in the Southern part of the country had (AOR= 1.75, 95%CI: 1.42–2.15) higher odds of attaining adequate PNC.

Women who had less than 4 times of ANC frequency had (AOR= 0.79, 95%CI: 0.62–0.85) smaller odds of having adequate PNC, as well as those with inadequate ANC quality (AOR= 0.56, 95%CI: 0.46–0.68), compared to those with 4 and more ANC frequency and adequate ANC quality, respectively. Moreover, those who had ANC in a public facility had (AOR= 0.57, 95%CI: 0.38–0.85) smaller odds of having adequate PNC compared to their private facility counterparts.

## Discussion

We assessed the content of PNC utilisation in Rwanda, as well as the associated socio-demographics using the 2020 RDHS dataset. In this study, five key components were used to assess adequate PNC utilisation, which included; having the cord examined, temperature of the baby measured, counselling on newborn danger signs, counselling on breastfeeding and having an observed breastfeeding session. To our knowledge, this is the first study to evaluate PNC content in Rwanda.

The study results indicated that less than half (44.3%) of the mothers had received all the above five PNC components, with cord examination, having breastfeeding session observed and counselling on breastfeeding being the most frequently reported (> 70%), but with less reporting (< 60%) for the baby’s temperature measurement and counselling on newborn danger signs. The observed prevalence of adequate PNC utilisation is still too low to achieve the desired reduction in postnatal-related child and maternal mortality and morbidity. This may be partly explained by a lack of sufficient community awareness regarding PNC components in Rwanda [23], or by the lower emphasis given to some PNC components by healthcare/PNC providers as highlighted by our results, and reported by *Kim* et al. [37]. Thus, there is a need for tailored ANC education to improve awareness of PNC contents among the mothers, as well as continued medical education for the various PNC providers. Nevertheless, such low rates of overall adequate PNC and imbalance in receipt of particular PNC components have also been reported in other resource-restricted countries such as; Burundi, Tanzania, Uganda, Mali, Nigeria, Zambia, Ethiopia, Nepal [38], Bangladesh [37], and rural China [39], amongst others.

The study also found several socio-demographics associated with receiving adequate PNC content, which included; exposure to newspapers/magazines, exposure to radio, parity, working status, visited by a fieldworker, distance to a health facility, region, permission to seek healthcare, ANC frequency, ANC quality, and ANC facility.

Women with no exposure to newspapers/magazines had less odds of receiving adequate PNC compared to the exposed ones, which might be because the unexposed women are more likely to miss out on the key maternal health and PNC information disseminated in the newspapers/magazines. However, women with no radio exposure interestingly were more likely to receive adequate PNC compared to those exposed to radio. There is no clear explanation for this finding, but it may be because of the propaganda or misinformation regarding healthcare delivery at government/public health facilities portrayed mainly via radio channels/programs. Nevertheless, although no study has explicitly evaluated the relationship between media exposure and PNC quality/content, media exposure has been shown to generally have a positive influence on PNC utilisation in other settings including Sierra Leone [5], Zambia [40], Southern Ethiopia [41], and Bangladesh [42].

Parity was also found to affect receipt of adequate PNC, whereby women with parity of less than 2 had less odds of getting adequate PNC compared to those with parity of 5 and above. This may be because women with multiple parities tend to have more experience regarding the importance of ANC and PNC acquired from previous pregnancies and births, unlike women giving birth for the first time [5]. Although this finding is in agreement with *Chakraborty* et al. [43], it deviates from several other studies that reported first-time mothers having more likelihood of complete PNC utilisation/ visits [44–48].

Working women also had less odds of having adequate PNC compared to non-working counterparts, which is probably since working mothers tend to have work duties/responsibilities as a competing priority and are more likely to miss out on PNC visits thus failing to achieve adequate PNC content. The finding is in line with several studies that have shown maternal working status as a barrier to complete PNC visits [5, 45, 47]. However, *Abebo* et al. reported that government-employed mothers in southern Ethiopia were more likely to receive complete PNC utilisation compared to other occupations [49]. This implies the need to enforce and strengthen favourable maternal leave policies, especially for women working in the private and informal sector, to enable them to achieve complete and adequate PNC utilisation.

Women visited by a fieldworker, were more likely to receive adequate PNC compared to the non-visited ones, possibly because fieldworkers tend to encourage and remind newborn mothers of the importance of adequate PNC utilisation thus achieving adequate PNC content. Not surprising this finding concurs with a PNC utilisation study that highlights visits by fieldworkers as an enabling factor [5].

Moreover, women having no big problem with distance to a health facility had higher odds of getting adequate PNC compared to their counterparts. Longer distances from a health facility hinder easy access to PNC services, leading to inadequate PNC content received by newborn mothers [48]. The finding concurs with PNC utilisation studies reporting distance as a hindering factor for access to maternal services [47, 48, 50, 51]. Region was also noted to be associated with adequate PNC, whereby women in the Southern part of the country were more likely to receive adequate PNC compared with those residing in the North. This could be partly explained by the higher altitude in the North, which may hinder access to health care facilities in some locations, resulting in a disparity in the utilization of health care facilities across the country, impeding access to PNC services. This supports previous research indicating altitude and overall geographic accessibility to health facilities as important predictors of maternal health services uptake [52, 53]. Other plausible factors include variability in cultural norms or religious beliefs in some parts of the country that disregard the importance of timely and adequate PNC utilization, for example, the practice of *Kwita Izina* which discourages the mother and her newborn from leaving the house until the baby is given a name, which is around the eighth day [13]. Nonetheless, our findings coincide with other studies which highlight regional imbalance as an associate of PNC utilisation in other low and middle-income countries [5, 48, 54, 55].

We expected the need for permission to seek healthcare to be a hindering factor for adequate PNC since it opposes women empowerment, as reported in several PNC utilisation studies [5, 48]. Contrary, the study results indicated that women with no big problem with permission to seek healthcare were less likely to receive adequate PNC compared to their counterparts having big problems with permission. Although no clear explanation, this observation could be a result of several mediating factors such as being visited by a fieldworker and a supportive family [51]. In this regard, women with permission problems, if identified earlier, would be more likely to be visited by a fieldworker or PNC providers at home, thus attaining adequate PNC. Another probable reason could be the reluctance of women with no permission problems knowing that they can go to a health facility anytime they want, unlike those with problems getting permission who might tend to ensure that when they are with a health worker, they get all the care needed considering it might be hard getting permission again.

Conjointly, women who had less ANC frequency and inadequate ANC quality were less likely to attain adequate PNC content compared to their respective counterparts. Apart from monitoring the pregnancy progress, ANC visits are also intended to educate, brief and counsel the mother on how to prepare for delivery and what to do during the postpartum and postnatal period [56, 57]. This implies that mothers with inadequate ANC quality are more likely to have missed the key PNC information including its importance, contents, when and where to receive it, thus having fewer chances of attaining adequate PNC. This implies a need for more focus on interventions aimed at increasing not only ANC frequency but also quality, including counselling on PNC contents, which has a multiplicative impact on increased PNC utilisation and quality [48]. Nonetheless, our findings concur with several studies that indicate ANC frequency and quality as enabling factors for PNC utilisation [5, 40, 45, 47, 49, 51].

Moreover, the type of health facility was also associated with adequate PNC, whereby women who had ANC in a public facility were less likely to attain adequate PNC compared to their private facility counterparts. The possible reason for this observation is because private health facilities tend to be less crowded with a smaller patient to doctor/midwife ratio and so more likely to provide better quality antenatal care with PNC follow-up [55]. Notably, although this finding agrees with *Dhaher* et al.*,* who reported higher PNC utilisation among women who delivered in private hospitals [55], there is generally inconclusive literature regarding the impact of health facilities (public vs. private) on PNC utilisation and quality, thus calls for a thorough investigation.

### Study strength and limitations

This is the first nationwide analysis that explores the content of postnatal care and the associated socio-demographic factors, and therefore, it can be used as a yardstick and motivation for further studies on the same topic in Rwanda and other countries. Additionally, we used the most recent nationally representative dataset, making our findings generalizable to all women in Rwanda. However, our study has some limitations worth acknowledging, including, the cross-sectional design which doesn’t allow the establishment of causal relationships, but rather only associations. The use of self-reported answers and the possibility of giving false answers due to social desirability risks recall and information bias. There was also a lack of data on other key determinants of adequate PNC such as male involvement and support, knowledge of PNC and the perceived quality of childbirth experience, counselling on PNC during ANC visits, among others, all of which could affect the uptake of PNC services.

## Conclusions

The study assessed adequate PNC and its associated factors, where it revealed that less than a half of the mothers in Rwanda had received adequate PNC contents, and of which cord examination, having breastfeeding session observed and being counselled on breastfeeding were the most performed. This is the first and most recent nationally representative study to provide data on the status of adequate PNC utilization in Rwanda. Hence crucial for the different maternal health stakeholders as they formulate policies and programmes aimed at achieving agenda 2030. With Rwanda performing well on utilization and access of care, there is an urgent need to focus on quality of care. The study results also provided a basic understanding of the socio-demographics associated with adequate PNC, which included; exposure to newspapers/magazines and radio, parity, working status, visited by a fieldworker, distance to a health facility, region, permission to seek health care, ANC frequency, ANC quality, and ANC facility. Utilization and quality of ANC has been shown to correlate strongly with access to adequate PNC hence a need to promote continuum of quality and easily accessible maternal care from antennal period to the postnatal period. Ministry of Health programs need to capitalize on existing positive interventions to further increase their coverage such as increasing airplay for maternal health programmes on radios and in newspapers, advocating for increase in maternal leave days to enable working women have adequate time to seek care and strengthening the community health programme to enable field health workers reach more women in the community which would mitigate the challenges of distance and seeking permission to access care. Furthermore, continued medical education and training of ANC and PNC providers, including the motivation of field health workers as well as reducing regional inequalities are all key in achieving adequate PNC utilisation and quality.

## Supplementary Information

**Additional file 1.**

## Data Availability

The data set used is openly available upon permission from the MEASURE DHS website (URL: https://www.dhsprogram.com/data/available-datasets.cfm). However, authors are not authorized to share this data set with the public but anyone interested in the data set can seek it with written permission from the MEASURE DHS website (URL: https://www.dhsprogram.com/data/available-datasets.cfm).

## Abbreviations

EA: Enumeration area
AOR: Adjusted Odds Ratio
CI: Confidence Interval
COR: Crude Odds Ratio
DHS: Demographic Health Survey
RDHS: Rwanda Demographic Health Survey
OR: Odds Ratio
SD: Standard Deviation
WHO: World Health Organization
ANC: Antenatal care
PNC: Postnatal care
SBA: Skilled Birth Attendance
SPSS: Statistical Package for Social Science
