# Supplementary material for: Prevalence of adequate postnatal care and associated factors in Rwanda: evidence from the Rwanda demographic health survey 2020
Source: Arch Public Health. 2022 Sep 16;80:208. doi: 10.1186/s13690-022-00964-6 (PMC9482265; doi:10.1186/s13690-022-00964-6)
Supplement: Supplementary file 1 — Additional file 1. [file 13690_2022_964_MOESM1_ESM.docx]

**Environmental factors**

-Health care systems

-Access to healthcare (distance)

**Outcome**

Adequate postnatal care utilization

**Population characteristics**

**-Predisposing factors**: Demographics (age, parity, education, marital status, region, household size, residence)

-**Enabling factors**: working status, health insurance, being visited by a field health worker, wealth index, exposure to mass media, permission and distance to seek healthcare

-**Need**: Perceived need for care: ANC Place, frequency and quality, place of childbirth

**Supplementary File 1:** Independent and outcome variables mapped on to the theoretical framework: Andersen’s Behavioral Model of Health Service Use.
